# Accurate estimation of pathway activity in single cells for clustering and differential analysis

**DOI:** 10.1101/gr.278431.123

**Published:** 2024-06

**Authors:** Daniel Davis, Avishai Wizel, Yotam Drier

**Affiliations:** The Lautenberg Center for Immunology and Cancer Research, IMRIC, Faculty of Medicine, Hebrew University of Jerusalem, Jerusalem 9112102, Israel

## Abstract

Inferring which and how biological pathways and gene sets change is a key question in many studies that utilize single-cell RNA sequencing. Typically, these questions are addressed by quantifying the enrichment of known gene sets in lists of genes derived from global analysis. Here we offer SiPSiC, a new method to infer pathway activity in every single cell. This allows more sensitive differential analysis and utilization of pathway scores to cluster cells and compute UMAP or other similar projections. We apply our method to COVID-19, lung adenocarcinoma and glioma data sets, and demonstrate its utility. SiPSiC analysis results are consistent with findings reported in previous studies in many cases, but SiPSiC also reveals the differential activity of novel pathways, enabling us to suggest new mechanisms underlying the pathophysiology of these diseases and demonstrating SiPSiC's high accuracy and sensitivity in detecting biological function and traits. In addition, we demonstrate how it can be used to better classify cells based on activity of biological pathways instead of single genes and its ability to overcome patient-specific artifacts.

Single-cell RNA sequencing (scRNA-seq) has become a staple technique in biomedical research, allowing for a deeper understanding of tissue characteristics and heterogeneity in health and disease (Chi and Deng 2020). Pathway analysis serves as a crucial tool for interpreting gene expression data, particularly in the context of scRNA-seq. Typically, this is accomplished by identifying a set of interesting genes, such as differentially expressed genes, and examining their enrichment within biological pathways. Although such approaches are very useful, there are many potential benefits to estimating pathway activity in each cell first and, only then, utilizing this information for downstream analysis. This overcomes limitations of single-cell data, such as inaccurate estimation of the expression of a single gene in a single cell, and enables the use of pathway activity for unsupervised analysis such as clustering.

Although methods for pathway analysis in bulk RNA-seq have been developed (Drier et al. 2013; Hänzelmann et al. 2013; Wang et al. 2016), they often prove inadequate for scRNA-seq data (Noureen et al. 2022). Past efforts to estimate pathway activity in single cells have used techniques such as AUCell (Aibar et al. 2017) and single-sample gene set enrichment analysis (ssGSEA) (Barbie et al. 2009). However, these methods were not designed for this type of analysis and are not always optimal.

Here we introduce single pathway analysis in single cells (SiPSiC), a new method tailored for analyzing the activity of a gene set or of a biological pathway, in single cells. SiPSiC achieves high sensitivity by relying on the normalized expression of all genes, weighted by their relative rank. Using SiPSiC, we reanalyzed scRNA-seq data of COVID-19, lung adenocarcinoma, and glioma; identified both known and novel cellular pathways involved in these diseases; and demonstrated SiPSiC's high accuracy and superior ability to identify changes in pathway activity missed by the original analyses. Furthermore, we propose new approaches for data clustering and visualization based on SiPSiC scores, mitigating biases inherent in scRNA-seq data and emphasizing functional similarity based on shared biological processes. Through comparative analyses with existing methods, we showcase SiPSiC's superior accuracy, sensitivity, and efficiency, positioning it as a valuable tool for pathway analysis in single-cell studies.

## Results

### A new tool to infer pathway activity in single cells

scRNA-seq data often suffer from sparsity owing to high dropout rates, posing challenges for accurate pathway analysis. To address this issue, we introduce SiPSiC, a new tool designed to calculate pathway scores for each individual cell and each gene set by using gene expression values normalized by the expression levels in other cells and weighted by the rank of the average gene expression across all genes of the gene set (see Methods). This robust weighted normalization enables accurate estimation of pathway activity, even in small data sets or when pathway gene coverage is limited.

Available on both GitHub and Bioconductor, SiPSiC is an easily installed and well-documented tool to dissect single-cell-level differences, allowing its users to interrogate tissue physiology and heterogeneity with high sensitivity and accuracy.

### SiPSiC reveals differential activity of pathways with potential therapeutic implications in SARS-CoV-2-infected cells

We applied SiPSiC to investigate changes in the activity of biological pathways after SARS-CoV-2 infection in two distinct data sets: single-nucleus RNA-seq data from recently deceased COVID-19 patients (Melms et al. 2021) and scRNA-seq of African green monkeys infected with SARS-CoV-2 or inactivated virus (Speranza et al. 2021). The original analysis of the human data included gene-based clustering from which cluster markers were inferred to elucidate cellular response to SARS-CoV-2 infection. The monkey data were originally analyzed by principal component analysis (PCA), clustering, and fast gene set enrichment analysis (Korotkevich et al. 2021). We calculated pathway scores per cell for each of the 50 MSigDB hallmark gene sets (Liberzon et al. 2015) and compared pathway scores between SARS-CoV-2 positive and negative controls. Our findings can be largely divided into three categories: (1) innate immune response of pneumocytes, (2) pathways modulated by the virus to support its life cycle, and (3) adaptive immune response in B and CD8^+^ T cells.

**Figure 1.**
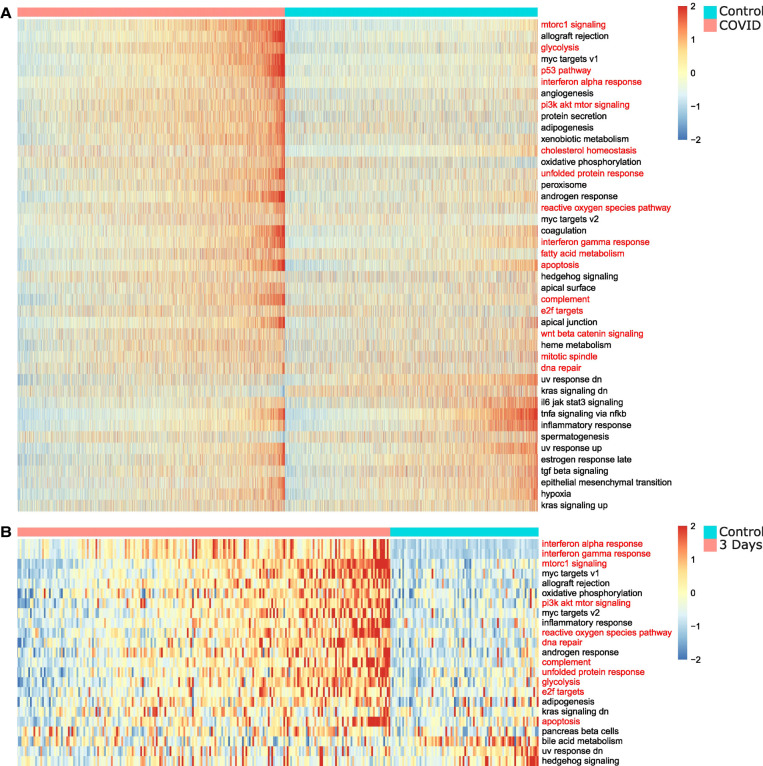
SiPSiC detects differential activity of hallmark pathways in SARS-CoV-2-infected alveolar cells. (*A*,*B*) Heatmaps depicting *Z*-scores of SiPSiC scores of alveolar cells for all differential hallmark pathways (FDR < 0.01) for COVID-19 patients and the control group (*A*) or SARS-CoV-2-infected monkeys and controls (*B*). Each row represents one hallmark pathway, and the pathway name is listed on the *right*. Pathways are sorted by significance of differential scores; cells in each cell group are sorted by their average *Z*-score across all pathways upregulated in that group. Pathway names mentioned in the text are colored red.

First, we applied SiPSiC to human alveolar cells (both type 1 and 2; 4575 cells from COVID-19 patients vs. 4303 control cells). Twelve out of 50 pathways were downregulated in COVID-19 patients, and 31 were upregulated (Student's *t*-test, FDR < 0.01) (Fig. 1A; see Supplemental Table S1). In addition to the interferon response, which was also reported as upregulated in alveolar type 2 (AT2) cells by Melms et al. (2021), we identified many other upregulated pathways involved in the innate immune response and its implications. Among them are genes involved in the complement pathway, DNA repair, and Wnt/beta catenin signaling. Indeed, the complement system was shown to be hyperactivated in severe SARS-CoV-2 infections (Afzali et al. 2022); SARS-CoV-2 induces DNA damage (Gioia et al. 2023); and WNT5A is upregulated in severe cases of COVID-19 (Choi et al. 2020). We also found upregulation of the mitotic spindle (FDR < 1.1 × 10^–4^) and E2F targets (FDR < 2.3 × 10^–15^), findings consistent with demonstrated hyperplasia of pneumocytes in humans and monkeys post SARS-CoV-2 infection (Ackermann et al. 2020; Speranza et al. 2021). In addition, Melms et al. (2021) found that the alveolar cells of the COVID-19 group showed lower expression of the ETV5 transcription factor required for maintaining AT2 cell identity compared with alveolar cells of the control lungs. Combined, these findings suggest that infected pneumocytes try to compensate for the damaged tissue by advancing the cell cycle and differentiating toward AT1 cells. Furthermore, SiPSiC detected upregulation of the hallmark apoptosis pathway (FDR < 1.1 × 10^–30^), consistent with detection of many apoptotic cells in human airway epithelium cultures infected with SARS-CoV-2 (Zhu et al. 2020). This may relate to apoptosis induction by the viral ORF3a protein (Ren et al. 2020). Additionally, it may relate to apoptosis induction by TP53, and indeed, SiPSiC also detected upregulation of the TP53 pathway ($\mathrm{FDR}<7.9\times 10^{-133}$). Prior research showed that viral infection upregulates TP53 by type 1 interferon signaling (Takaoka et al. 2003) and, on the other hand, that TP53 both activates the interferon pathway and promotes type 1 interferon release from cells undergoing viral infection (Muñoz-Fontela et al. 2008). These findings together suggest this positive feedback loop of interferon and TP53 may also play a role in SARS-CoV-2 infection.

In the category of pathways modulated by the virus to support its life cycle, SiPSiC found upregulation of the PI3K/AKT/MTOR and mTORC1 pathways, consistent with evidence that these pathways are activated after SARS-CoV-2 infection (Appelberg et al. 2020). Moreover, mTORC1 activation is known to induce expression of key enzymes involved in several metabolic pathways, including glycolysis and biosynthesis of fatty acids and cholesterol (Düvel et al. 2010), supporting SiPSiC's findings that glycolysis, fatty acid metabolism, and cholesterol homeostasis were all upregulated in the alveolar cells of COVID-19 patients. Further support for the upregulation of glycolysis is provided by previous works showing that SARS-CoV-2 infection indeed increases glycolysis, both in colon carcinoma cells and in monocytes (Bojkova et al. 2020; Codo et al. 2020). mTORC1 has also been shown to activate the IRE1-JNK signaling pathway of the unfolded protein response (UPR), thereby triggering apoptosis (Kato et al. 2012). SiPSiC found that the UPR pathway was upregulated, too ($\mathrm{FDR}<1.16\times 10^{-68}$), consistent with the activation of mTORC1 and previous research suggesting that SARS-CoV-2 induces the UPR (Echavarría-Consuegra et al. 2021). A previous review stated that UPR activation can increase type 1 interferon production (Sprooten and Garg 2020). Combined, all these findings suggest that SARS-CoV-2 infection increases endoplasmic reticulum (ER) stress and UPR activation, which are also enhanced by the viral-induced upregulation of mTORC1, thereby promoting apoptotic cell death and possibly also the positive feedback loop involving TP53 and type 1 interferons, which further encourages apoptosis.

Another pathway in this category found upregulated consists of genes upregulated by reactive oxygen species (ROS). Oxidative stress is known to be induced by several viruses (Lee 2018), and monocytes infected with SARS-CoV-2 had a higher production of mitochondrial ROS (Codo et al. 2020), suggesting that SARS-CoV-2 also induces oxidative stress in infected cells. Together, these results suggest that SARS-CoV-2 activates MTOR and induces glycolysis and oxidative stress. We applied SiPSiC to human activated B cells (55 cells of COVID-19 patients, 48 control cells) and CD8^+^ T cells (103 COVID-19 cells, six control) to better characterize the adaptive immune response. Despite the relatively small cell populations, TGF beta signaling was found upregulated in activated B cells of COVID-19 patients (FDR < 0.006), consistent with previous reports (Ferreira-Gomes et al. 2021; Melms et al. 2021). The G_2_/M checkpoint pathway was upregulated in the CD8^+^ T cell group of COVID-19 patients (FDR < 0.003), a finding missed by the conventional analysis in Melms et al. (2021) but consistent with the expected increase in T cell proliferation in the lungs (Liao et al. 2020). The interferon gamma (IFNG) response pathway was found upregulated in the SARS-CoV-2-infected CD8^+^ T cells as well, albeit with borderline statistical significance (FDR < 0.031). This finding correlates well with prior evidence of elevated interferon levels in the plasma of COVID-19 patients and the resulting effect on immune cells, as well as with previous evidence showing upregulation of interferon-related genes across different types of immune cells collected from the mediastinal lymph nodes of SARS-CoV-2-infected monkeys (Schultheiß et al. 2020; Speranza et al. 2021).

Many of the pathways that SiPSiC found to be upregulated in the alveolar cells of the COVID-19 group were of therapeutic potential, including DNA repair, UPR, PI3K/AKT/MTOR signaling, ROS, and metabolic pathways. Drugs targeting the DNA damage response can block SARS-CoV-2 replication (Garcia et al. 2021), suggesting that SARS-CoV-2 not only increases DNA damage in the infected cells but also relies on the cells’ reaction to it. SARS-CoV-2 replication can be blocked by UPR inhibitors (Echavarría-Consuegra et al. 2021), PI3K/AKT/MTOR inhibitors (Appelberg et al. 2020; Klann et al. 2020; Stukalov et al. 2021; Yuen et al. 2021), and drugs targeting lipid metabolism (Abu-Farha et al. 2020; Williams et al. 2021). Treatment of COVID-19 patients with *N*-acetylcysteine (NAC), a precursor of the antioxidant agent glutathione, correlated with lower mortality in a retrospective study (Izquierdo et al. 2022), and SARS-CoV-2-infected monocytes treated with antioxidant agents such as NAC showed reduction in both viral replication and production of several cytokines, particularly interferon type 1 and 2 (Codo et al. 2020). Codo et al. (2020) further showed that the increased production of ROS in infected monocytes promoted glycolysis and that inhibitors of glycolysis also reduced SARS-CoV-2 replication and could reduce the production of type 1 and 2 interferons. Notably, glycolysis inhibitors also reduced SARS-CoV-2 replication in colon carcinoma cells (Bojkova et al. 2020).

Together these findings suggest that MTOR signaling and its impact on ROS and metabolism play an important role in the pathophysiology of COVID-19, and drugs targeting several of these pathways could have synergistic effects with consequences on disease progression and severity.

Analysis of the African green monkey COVID-19 data set supported many of our findings from the human COVID-19 data set, including pathways from all three categories. Differentially expressed pathways detected in the analysis of the alveolar cells are presented in Figure 1B. In addition, SiPSiC analysis of the immune cells from the monkeys’ lungs revealed additional pathways involved in the response of lymphocytes to SARS-CoV-2 infection. Complete results of this data set can be found in Supplemental Note 1 and Supplemental Table S2.

### SiPSiC detects key activated pathways in a tumor-specific epithelial lineage of lung adenocarcinoma

To further demonstrate SiPSiC's robustness, we applied it to lung adenocarcinoma (Kim et al. 2020) and compared three malignant epithelial lineages denoted by Kim et al. (2020) as tS1, tS2, and tS3 (5880 cells total, including 2879 tS1, 2938 tS2, and 63 tS3 cells) (Supplemental Table S3). Whereas tS1 and tS3 have healthy epithelial counterparts, tS2 showed a tumor-specific phenotype; hence, we focused on differentially active pathways in this lineage (Supplemental Fig. S1A). In line with the findings of Kim et al. (2020), the apoptosis (FDR < 2.4 × 10^–3^) and late response to estrogen (FDR < 3.3 × 10^–4^) pathways were found upregulated in tS2 cells compared with both tS1 and tS3. In addition, SiPSiC found upregulation of the hypoxia and glycolysis pathways in tS2 cells (FDR < 1.3 × 10^–6^, FDR < 3.2 × 10^–12^), suggesting hypoxic conditions drive glucose metabolism in tS2 cells. Indeed, a lung adenocarcinoma hypoxia signature (Mo et al. 2020) is upregulated in tS2 cells, supporting our findings. Of note, Mo et al. (2020) reported that high expression of this signature was correlated with higher infiltration of activated CD4^+^ T cells and M0 macrophages, whereas Kim et al. (2020) showed that a positive correlation exists between the proportions of tS2 cells and exhausted CD8^+^ T cells or monocyte-derived macrophages. Indeed, SiPSiC found upregulation of several inflammatory pathways in the tS2 cells, including the inflammatory response, complement, allograft rejection, and IL6/JAK/STAT3 signaling, suggesting an immune response in tS2 cells and agreeing with immune cell infiltration.

Kim et al. (2020) showed that higher expression of the tS2-specific genes is correlated with metastasis and poorer prognosis. Several pathways SiPSiC identified as upregulated may be involved, including PI3K/AKT/MTOR (FDR < 2.9 × 10^–230^ and FDR < 0.019 compared with the tS1 and tS3 lineages, respectively), mTORC1 (FDR < 1.4 × 10^–11^), and epithelial-to-mesenchymal transition (EMT; FDR < 7.7 × 10^–10^), previously demonstrated to be involved in lung adenocarcinoma metastasis (Krencz et al. 2017; Ding et al. 2018; Lu et al. 2020). In addition, cell cycle pathways (G_2_/M checkpoint, FDR < 3.3 × 10^–4^; E2F targets, FDR < 4.4 × 10^–4^) are upregulated, suggesting tS2 cells proliferate faster and can therefore contribute to tumor aggressiveness.

### SiPSiC analysis of glioma reveals novel differentially active pathways showing potential therapeutic implications

To validate the applicability of SiPSiC to different data types, we analyzed scRNA-seq data of glioblastoma tumors (Neftel et al. 2019). In their work, Neftel et al. (2019) identified four malignant “metamodules” (cellular states): oligodendrocyte-progenitor-like (OPC-like), neural-progenitor-like (NPC-like), astrocyte-like (AC-like), and mesenchymal-like (MES-like). We calculated pathway scores per cell (n = 6576) for each of the same 50 hallmark pathways. The cells were then split into four groups based on their cell state assignments (1986 NPC-like, 1047 OPC-like, 1929 AC-like, and 1614 MES-like cells), and comparisons were made between each pair of groups (Supplemental Table S4; Supplemental Fig. S1B). We found that the G_2_/M checkpoint pathway was upregulated in the OPC- and NPC-like groups compared with the AC- and MES-like groups, indicative of a higher proportion of proliferating cells in these cell states. In addition, the hypoxia response pathway was enriched in the MES-like group compared with all other three groups. These findings are consistent with the findings reported in Neftel et al. (2019). Furthermore, SiPSiC analysis suggests that the MES-like group was enriched in the inflammatory response pathway, in concordance with prior evidence that the mesenchymal subtype of GBM tumors is enriched in inflammatory response–associated genes (Engler et al. 2012).

Notably, SiPSiC analysis of the glioblastoma data set also indicated pathways with therapeutic implications. The TGF beta (FDR < 4.86 × 10^–14^), TNFA via NF-kB (FDR < 2.77 × 10^–117^), and IL6/JAK/STAT3 (FDR < 8.86 × 10^–36^) signaling pathways and the EMT pathway (FDR < 1.02 × 10^–113^) were all found upregulated in the MES-like group. A previous work has shown that in gliomas, regulatory T cells secrete TGFB1 and thereby promote the NF-kB-IL6-STAT3 signaling axis, and IL6 receptor blockers have a potential therapeutic effect (Liu et al. 2021). TGF beta signaling also activates TNF signaling via NF-kB in glioblastoma, which in turn induces mesenchymal transition (Bhat et al. 2013; Yan et al. 2022). Furthermore, these works also suggest that these two pathways can be targeted to improve overall survival and specifically attenuate resistance to radiotherapy in glioblastoma patients. Of relevance, the mesenchymal subtype of glioblastoma is correlated with high levels of both immune markers and infiltration of immune cells (Verhaak et al. 2010; Wang et al. 2017). Together, these findings suggest that regulatory T cells infiltrate glioblastoma tumors, promoting the TGF beta, TNF via NF-kB, and IL6/JAK/STAT3 signaling pathways and thereby increasing mesenchymal transition of tumor cells.

Furthermore, SiPSiC detected upregulation of the KRAS signaling up ($\mathrm{FDR}<9.56\times 10^{-22}$) and downregulation of the KRAS signaling down ($\mathrm{FDR}<1.7\times 10^{-8}$) pathways in the MES-like group, indicative of KRAS signaling activation in this group. These findings are consistent with the finding that RAS and TGFB1 cooperate to induce EMT in epithelial cells and evidence that KRAS activation in glioblastoma cells induces a mesenchymal shift (Kim et al. 2014; Marques et al. 2021; Zhao et al. 2021). SiPSiC also identified upregulation of ROS in the MES-like group compared with all other groups ($\mathrm{FDR}<1.05\times 10^{-57}$), suggesting that the promotion of TGFB1-induced EMT by RAS is mediated by enhanced production of ROS, as was shown in mammary epithelial cells (Kim et al. 2014). Taken together, our findings suggest a synergistic effect in glioblastoma in which regulatory T cells activate TGFB1 signaling, leading to EMT, which is enhanced by RAS activation. Additionally, RAS inhibition was shown fatal to glioblastoma cells (Blum et al. 2005). Hence, our analysis suggests that a combined therapy targeting RAS and TGFB1 or their downstream targets could have a synergistic therapeutic effect for glioblastoma, again demonstrating the potential of SiPSiC analysis to accelerate the development of targeted therapy.

Garofano et al. (2021) suggested a different assignment of the cells to four clusters based on metabolic and other functional characteristics (Garofano et al. 2021), and reported the results of pathway analysis for these clusters. Our analysis was found highly consistent with their results, with 95% of the hallmark pathways reported by them similarly detected by SiPSiC (see Supplemental Note 2).

To further validate SiPSiC, we applied it to an oligodendroglioma data set (Tirosh et al. 2016). Here too, SiPSiC analysis was consistent with the findings reported by Tirosh et al. (2016), whereas it also allowed us to detect pathways not reported in the original analysis (for the complete results, see Supplemental Note 3; Supplemental Table S5; Supplemental Fig. S1C).

To conclude, SiPSiC differential pathway analyses of the COVID-19 and glioma data sets demonstrate that SiPSiC can accurately detect key differentially active pathways even in sparse data sets with few cells or when many of the pathways’ genes are not available in the data. This allows for comprehensive and sensitive pathway analysis in single cells, which can serve to study fundamental biology as well as generate clinically and therapeutically relevant hypotheses that may be missed by standard analysis.

### Unsupervised clustering by SiPSiC scores identifies shared biological features and cellular identities

Unsupervised clustering of scRNA-seq data sets allows the demarcation of distinct cell subpopulations within tissues, offering insights into tissue heterogeneity in various scenarios. To assess the potential benefits of clustering based on SiPSiC pathway scores compared with the conventional approach of clustering by gene expression, we calculated cell clusters and uniform manifold approximation and projection (UMAP) for 9635 malignant glioblastoma cells (Neftel et al. 2019), both by gene expression and by SiPSiC scores (Methods).

**Figure 2.**
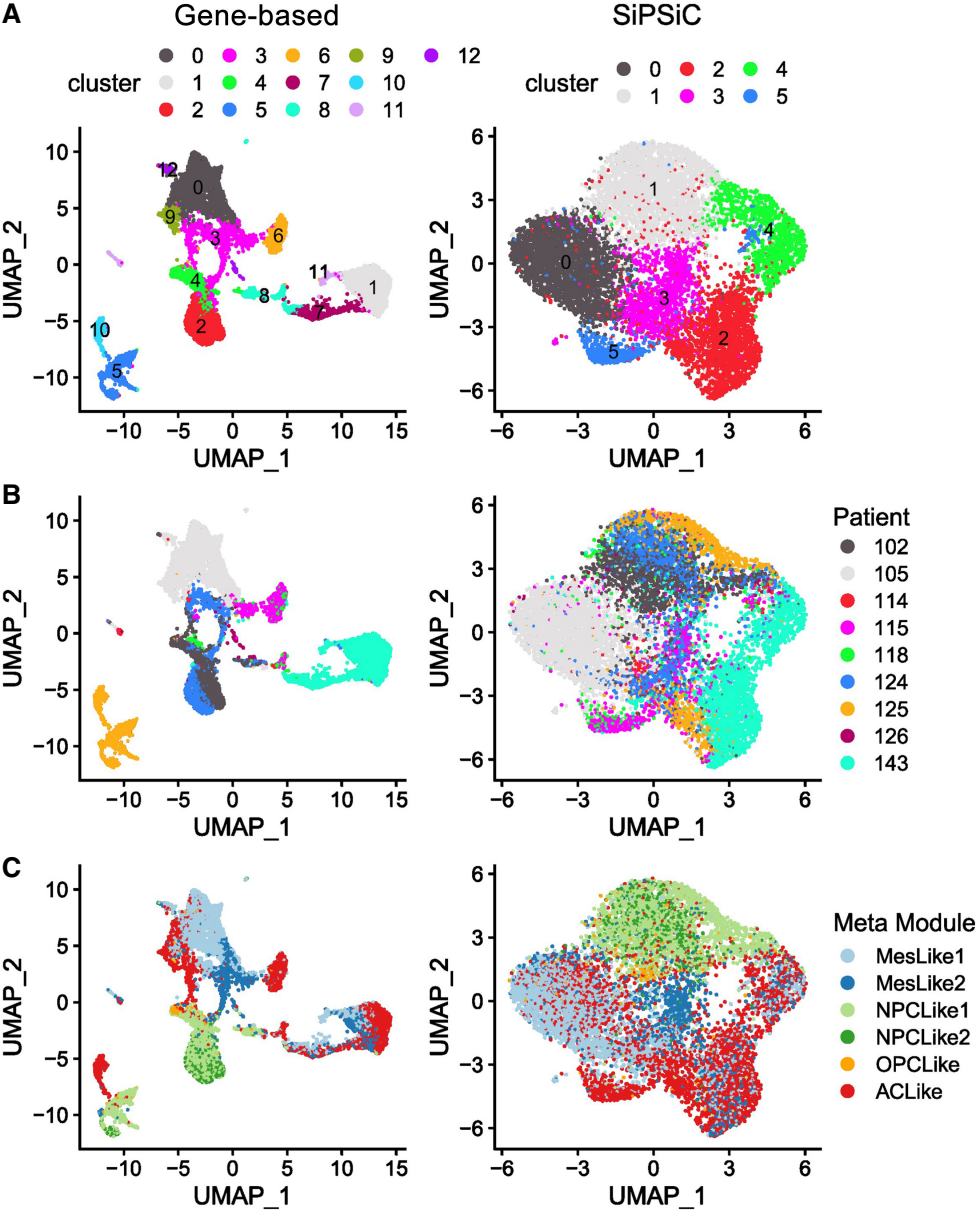
SiPSiC-based clustering overcomes patient biases presented by gene-based clustering. (*A*–*C*) UMAP projections based on gene expression (*left*) and hallmark pathway scores by SiPSiC (*right*). (*A*) Cells were clustered by Louvain algorithm according either to SiPSiC scores or to gene expression. UMAPs show cells colored by cluster. (*B*) Cells colored by patient identity. (*C*) Cells colored by malignant metamodule assignment.

Gene-based clustering produced 13 clusters. Although the data set was sampled from nine different patients, in five clusters (0, 1, 5, 9, and 10) >99% of the cells were of a single patient, and in one other (cluster 7), 97% of the cells were of a single patient, reflecting the strong patient-bias typical for gene-based clustering of malignant cells (Fig. 2A,B). In contrast, using SiPSiC pathway scores for clustering with the same algorithm and resolution produced six clusters. In five of these clusters no more than 76% of the cells were of a single patient, suggesting that patient-specific batch effects have limited effect on SiPSiC-based clustering (Fig. 2A,B). Although 96% of the cells in cluster 0 belong to patient 105, most of these cells are classified as MES-like1 cells, suggesting this cluster may capture their MES-like1 identity and not necessarily patient-specific artifacts. Together, this suggests that SiPSiC-based clustering was largely based on similar biologically relevant features of the cells rather than patient-specific batch effects.

Clustering by SiPSiC scores also allows the detection of functional heterogeneity in an unsupervised manner by comparing differential pathway scores between the clusters (see Supplemental Table S6). Cluster 1 was enriched in the Wnt/beta catenin and hedgehog signaling pathways, both found upregulated in the NPC-like metamodule in our differential pathway analysis of the Smart-seq2 glioblastoma data set above (Supplemental Fig. S1B). Indeed, 2096 (84%) out of the 2496 cells in this cluster were NPC-like cells (Fig. 2C). Furthermore, the adjacent cluster 4 contains 339 additional NPC-like cells, forming a distinct group of 2435 (96.4%) of the 2527 NPC-like cells in the data on the UMAP projection. Similarly, 12 out of the 13 pathways found enriched in cluster 3 were detected by SiPSiC as upregulated in the MES-like cells, and 883 (76%) out of the 1164 cells in this cluster are MES-like cells. The adjacent clusters 0 and 2 contain 1977 and 644 additional MES-like cells, respectively, forming together with cluster 3 a large group of 3504 (87%) of the total 4017 MES-like cells on the UMAP projection (Fig. 2C). Moreover, three out of the four pathways that were enriched in cluster 5 showed upregulation in AC-like cells: The interferon alpha and angiogenesis pathways are significantly upregulated in the AC-like cells versus all other metamodules, as well as the IFNG pathway that is significantly upregulated compared with NPC- and OPC-like cells (Supplemental Fig. S1B; Supplemental Table S4). Indeed, 356 (78%) of the 455 cells in this cluster are AC-like cells (Fig. 2C).

In contrast, testing the gene-based clusters for enrichment of these cellular identities, we found that both the MES-like and NPC-like cells were split across different groups of clusters. Although clusters 0, 3, and 9 contain 2754 (69%) of the MES-like cells in the data, additional 955 (24%) MES-like cells are found in clusters 1, 7, and 11 (Fig. 2A,C). Of note, <21% of the cells in cluster 9 are MES-like cells. NPC-like glioblastoma cells are even more scattered across different clusters. Although 1680 (66%) of the NPC-like cells are found in clusters 2 and 4, clusters 5 and 8 contain an additional 570 (23%) and 170 (7%) NPC-like cells, respectively, resulting in three clearly distinct groups of NPC-like cells on the UMAP (Fig. 2A,C); 99.8% of the cells in cluster 5 belong to a single patient, again showing strong patient bias that hinders the ability to cluster the cells properly.

Together, these results demonstrate that SiPSiC scores emphasize common cellular functions, are robust to batch effects, and are therefore better fitted to uncover the biological underpinnings of different cell clusters. Other methods were developed to explicitly remove known batch effects such as patient-specific differences. To compare SiPSiC clustering results to explicit patient batch correction, we applied Seurat integration (Butler et al. 2018), Harmony (Korsunsky et al. 2019), and scVI (Lopez et al. 2018) to the same data set and repeated the clustering pipeline (Supplemental Fig. S2). All three methods removed the differences between patients, similarly revealing the functional clustering discovered by SiPSiC. Therefore, although we do not suggest SiPSiC as a batch correcting tool per se, we do point to its utility in focusing on biologically relevant cellular characteristics. Moreover, it can be useful even in the context of overcoming artifacts, especially when the batches and the source of the artifacts are unknown and cannot be directly removed or when batch correction overcorrects real biological differences.

### Benchmarking of pathway scoring methods reveals higher accuracy and improved scalability of SiPSiC for different data types

To compare SiPSiC results to state-of-the-art methods for scoring pathway activity in single cells, we repeated the differential pathway analyses of the glioblastoma and both COVID data sets with AUCell (Aibar et al. 2017), variance-adjusted Mahalanobis (VAM) (Frost 2020) and ssGSEA (Barbie et al. 2009).

**Figure 3.**
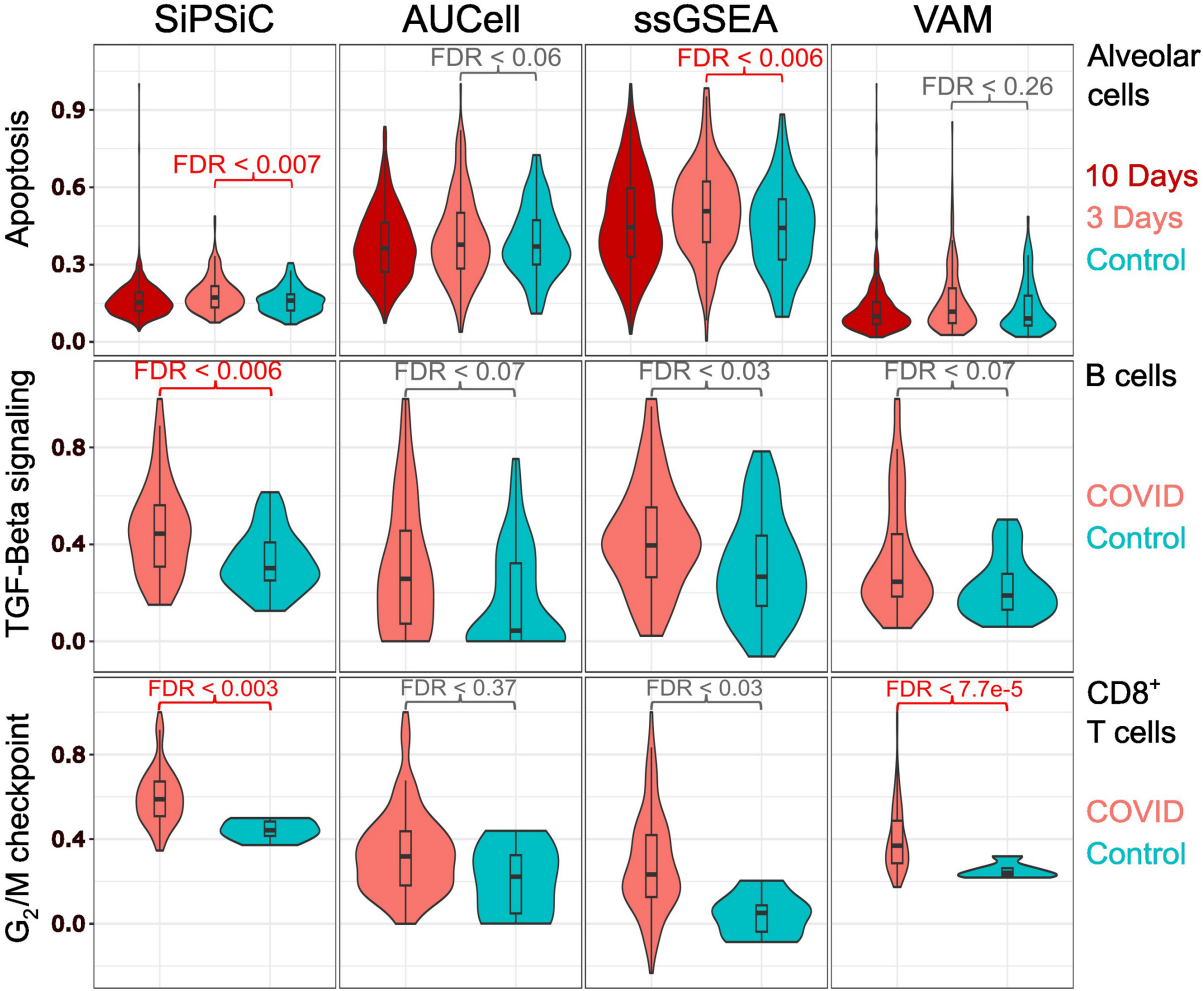
SiPSiC shows improved accuracy compared with AUCell, ssGSEA, and VAM. Violin plots showing normalized pathway score distributions as calculated by the four different methods. Significant results (FDR < 0.01) are colored red. (*Top*) Apoptosis, monkey alveolar cells. (*Middle*) TGF beta signaling, human B cells. (*Bottom*) G_2_/M checkpoint, human CD8^+^ T cells.

A summary of differentially active pathways between the actively infected monkeys and controls by all four methods can be found in Table 1. All four methods found upregulation of the mTORC1 signaling, MYC targets V1, oxidative phosphorylation, ROS, allograft rejection, and both interferon pathways in the active infection group of the alveolar cells. However, several differences were observed between the different methods. SiPSiC and ssGSEA were the only methods to detect significant upregulation of apoptosis (Fig. 3), whereas SiPSiC was the only method to detect upregulation of the adipogenesis pathway in alveolar cells. Both findings are supported by all four methods detecting the same pathways in the active infection group of the human alveolar cells.

**Table 1.** Summary of the number of differentially active pathways in the active infection group of the COVID monkey data set, by all four tested methods

Cell type	Pathway status	SiPSiC	AUCell	ssGSEA	VAM	Overlap
Alveolar	Upregulated	31	30	34	28	24
	Downregulated	12	9	8	10	4
	Skipped	0	4	0	0	0
	Missed	0	4	1	3	—
B cells	Upregulated	1	0	0	0	0
	Downregulated	0	0	0	0	0
	Skipped	0	19	0	0	0
	Missed	0	0	0	0	—
T cells	Upregulated	1	0	1	3	0
	Downregulated	0	0	0	0	0
	Skipped	0	24	0	0	0
	Missed	0	0	0	0	—

The overlap column shows the number of pathways found as differentially active by all methods. Skipped pathways are pathways for which AUCell could not calculate scores, and missed pathways are cases in which the relevant method failed to detect differential activity of a pathway detected by all other methods. For the analysis of all 50 hallmark pathways, see Supplemental Table S2.

To compare the methods in an unbiased manner, we defined misses of a method as pathways whose differential activity was consistently detected by all three other methods (FDR < 0.01) but not by the method in question (either did not pass FDR or changed in the opposite direction). For instance, AUCell failed to detect differential activity of the complement pathway (FDR < 0.5) in the active infection group of the monkey alveolar cells, whereas all the other methods showed significant upregulation. AUCell also failed to detect differential activity of the mitotic spindle, interferon alpha, and interferon gamma pathways in B cells, whereas all other methods found upregulation of the interferon pathways and downregulation of the mitotic spindle pathway, suggesting SARS-CoV-2 infection hinders B cell proliferation in the host's lungs.

Comparing the results of the CD8^+^ T cell analyses, we found that all methods successfully detected the activation of cells, as all four showed upregulation of the TNFA via NF-kB, complement, inflammatory response, and interferon pathways in this group. However, SiPSiC was the only method to detect upregulation of both MYC target pathways in the active infection group, whereas AUCell missed the MYC target V2 pathway, producing a median score of zero for all T cell groups, and the two other methods failed to reach statistical significance for the MYC target V1 pathway (Supplemental Fig. S3). As mentioned above, CD8^+^ T cell proliferation can be expected in the lungs of COVID-19 patients, and a significantly higher (89%) average *MYC* expression was indeed found in the active infection group compared with the control in this data set (*P* < 0.0032, unpaired Wilcoxon test), supporting SiPSiC's higher robustness.

For the human COVID data set, we found the methods yielded similar results regarding the upregulated pathways in alveolar cells of COVID-19 patients, with 24 pathways detected by all four (Table 2). The inflammatory response, IL6/JAK/STAT3 signaling, and TNFA signaling via NF-kB were all downregulated according to all four methods, suggesting a counterintuitive reaction of infected epithelial cells suppressing immune response. Additionally, the TGF beta signaling pathway was found downregulated by all methods except ssGSEA, providing further support for this putative anti-inflammatory response. In activated B cells, SiPSiC was the only method that managed to show upregulation of TGF beta signaling (Fig. 3), consistent with previous reports as mentioned above, whereas all other methods did not find any differentially active pathway in this group. Similarly, SiPSiC and VAM were the only methods to detect upregulation of the G_2_/M checkpoint pathway in CD8^+^ T cells (Fig. 3), in line with the upregulation of *MYC* found in the monkey T cells and the expected proliferation of these cells as mentioned above.

**Table 2.** Summary of the number of differentially active pathways in COVID patients, by all four methods

Cell type	Pathway status	SiPSiC	AUCell	ssGSEA	VAM	Overlap
Alveolar	Upregulated	20	9	18	11	7
	Downregulated	3	10	9	0	0
	Skipped	0	2	0	0	0
	Missed	0	3	1	2	—
B cells	Upregulated	2	0	4	2	0
	Downregulated	7	0	6	2	0
	Skipped	0	20	0	0	0
	Missed	0	4	0	0	—
T cells	Upregulated	20	12	17	11	7
	Downregulated	4	4	6	0	0
	Skipped	0	16	0	0	0
	Missed	0	3	0	2	—

The overlap column shows the number of pathways found as differentially active by all methods. Skipped pathways are pathways for which AUCell could not calculate scores, and missed pathways are cases in which the relevant method failed to detect differential activity of a pathway detected by all other methods. For the analysis of all 50 hallmark pathways, see Supplemental Table S1.

For the glioblastoma data, we found less agreement between the methods (see Table 3). SiPSiC showed no misses, demonstrating its higher robustness across data types; however, AUCell missed three pathways and ssGSEA and VAM missed five pathways each, with most misses owing to biases toward specific metamodules. VAM showed bias toward the MES-like group (28 upregulated pathways compared to 20, 23, and 17 detected by AUCell, SiPSiC, and ssGSEA, respectively) and NPC-like group (13 upregulated pathways compared to six, eight, and four detected by AUCell, SiPSiC, and ssGSEA, respectively). Specifically, VAM detected upregulation of the adipogenesis pathway in the MES-like group, whereas all other methods found it was upregulated in the AC-like group. This bias prevented VAM from detecting differential pathway activity in either the MES- or NPC-like group over the other, which accounts for three more misses of the method. Indeed, although all other methods found upregulation of the PI3K/AKT/MTOR signaling and heme metabolism pathways in the MES-like group and found upregulation of the hedgehog signaling in the NPC-like group, VAM detected upregulation of the PI3K/AKT/MTOR signaling pathway in the NPC-like group, whereas it failed to achieve statistical significance comparing the NPC- and MES-like groups in the two other pathways. In summary, these biases account for four out of the five misses of VAM.

**Table 3.** Summary of the number of differentially active pathways in each glioblastoma metamodule, according to the four tested methods

	SiPSiC	AUCell	ssGSEA	VAM	Overlap
AC-like	4	13	10	1	1
MES-like	23	20	17	28	13
OPC-like	2	2	10	0	0
NPC-like	8	6	4	13	2
Missed	0	3	5	5	—

Missed pathways are cases in which the relevant method failed to detect differential activity of a given pathway in the specific cell group where all other methods detected the pathway. For a summary of the analysis of all 50 hallmark pathways, see Supplemental Table S4.

ssGSEA, in contrast, showed bias toward the OPC-like group, with 10 pathways detected as upregulated compared to two, two, and none detected by AUCell, SiPSiC, and VAM, respectively. This bias accounts for four out of ssGSEA's five misses, as it found upregulation of the Wnt/beta catenin, G_2_/M checkpoint, and E2F targets pathways in the OPC-like group—all found upregulated in the NPC-like group by the other methods—and failed to show significant difference between the OPC- and MES-like groups in the mTORC1 signaling pathway, which was found upregulated in the MES-like group by all other methods. A similar bias was presented by AUCell toward the AC-like group with 13 pathways found upregulated compared to four, 10, and one found by SiPSiC, ssGSEA, and VAM, respectively. This bias accounts for two of AUCell's three misses, one of them being the inflammatory response pathway, which all other methods detected as upregulated in the MES-like group, consistent with previous knowledge as mentioned above.

Of note, SiPSiC was the only method with no misses across all cell types in all three data sets, suggesting it is the most robust method of the four (see Tables 1–3).

We also compared the execution time of the different methods, applied to the same three data sets (see Methods). The detailed execution times and fold change figures compared with SiPSiC can be found in Supplemental Table S7. AUCell, ssGSEA, and VAM were respectively at least 43%, 169%, and 77% slower than SiPSiC across all cell types and data sets, excluding the B cells of monkeys, for which AUCell was slightly quicker than SiPSiC. On average, we saw a fold change of 1.99, 11.06, and 3.71 in the execution times of AUCell, ssGSEA, and VAM across the data sets compared with SiPSiC execution times. In addition, the differences in execution times were largely correlated with data set size so that SiPSiC's advantage was greater in larger data sets. For instance, although AUCell, ssGSEA, and VAM showed a fold change of 0.77, 2.69, and 1.77 compared with SiPSiC for the B cells of monkeys (113 cells), these numbers increased to 1.85, 9.25, and 2.33, respectively, in the analysis of monkey alveolar cells, in which 1004 cells were included. Moreover, in the glioblastoma analysis (6576 cells), SiPSiC executed in just 5.7 sec, whereas AUCell, ssGSEA, and VAM took 14.1 sec (247% of SiPSiC's execution time), 146.2 sec (2565%), and 20.5 sec (360%) to complete. Therefore, SiPSiC is more scalable and better fitted for use on large data sets, allowing meta-analysis of large amounts of single-cell data becoming available now and in the near future.

### SiPSiC is robust to normalization parameter settings

SiPSiC normalizes the expression of each gene by the median expression level of the top τ% of cells (see Methods). The above analysis was conducted with the default value of τ = 5%. To test the robustness of SiPSiC results to τ, we repeated the analyses of the same five data sets with τ = 2% and τ = 10% (Supplemental Table S8). The different values of τ had only minimal impact on the results, and most pathways were either found not significantly different or found significantly upregulated in the same group of cells. In the lung adenocarcinoma and both COVID data sets, between 46 (92%) and 49 (98%) of the 50 hallmark pathways produced the same results across all comparisons. For glioblastoma and oligodendroglioma, 38 (76%) and 43 (86%) of the pathways produced the same result.

Across the data sets, we found only three pathways in which results with τ = 5% were inconsistent with both τ = 2% and τ = 10%. These are the IFNG pathway in the glioblastoma analysis, the allograft rejection in the T cells of monkeys, and the genes downregulated by UV in T cells of human SARS-CoV-2 patients. In glioblastoma, upregulation of the IFNG response in AC-like cells was observed with τ = 2% and in MES-like cells with τ = 10%, whereas with τ = 5%, both groups were found upregulated compared with OPC-like and NPC-like cells as part of an overall bias toward AC-like cells upregulation with τ = 2% and MES-like cells with τ = 10% (Supplemental Table S8). For the allograft rejection pathway changes were minimal but just crossed the selected threshold of FDR < 0.01 (0.0094 for τ = 2%, 0.0112 for τ = 5%, and 0.0089 for τ = 10%). For genes downregulated by UV, downregulation in T cells was detected with τ = 2% and τ = 10% but was not significant with τ = 5%, as well as according to AUCell, ssGSEA, and VAM, suggesting the results with τ = 5% better reflect reality.

## Discussion

scRNA-seq is a powerful technique for interrogating cellular heterogeneity, allowing researchers to comprehensively query changes in biological processes in high resolution. Despite its utility, computational methods for inferring the activity of biological processes are still lacking. As the scale, resolution, and abundance of scRNA-seq continue to grow, there is a pressing need to refine existing methodologies and develop novel improved ones. These endeavors are pivotal for uncovering novel biological processes and elucidating poorly understood phenomena.

In this paper, we introduce SiPSiC, a novel method for inferring pathway scores from scRNA-seq data. We demonstrate its applicability and high sensitivity, accuracy, and consistency by applying it to publicly available data sets of COVID-19 and various malignancies. Our analyses reveal numerous pathways exhibiting altered activity, many of which were not previously identified in the original papers. These findings align with established biological knowledge and are corroborated by other research studies, underscoring the biological relevance of SiPSiC-derived insights.

SiPSiC presents several advantages over the conventional approach of gene set enrichment of differential genes. Its primary benefit lies in its capability to assess pathway activity for every single cell, allowing more robust detection of changes in pathway activity, estimation of heterogeneity in pathway activity changes, and representation of the data in pathway space. We suggest a novel approach for dimensional reduction and clustering for single-cell data using SiPSiC scores rather than gene expression profiles. This strategy accentuates biological similarities between cells, while mitigating technical artifacts and covariates such as patient of origin. Although SiPSiC is not a batch correction method, it offers distinct advantages that may render batch correction unnecessary in certain scenarios. Unlike batch correction methods, SiPSiC does not artificially alter the raw data, thus avoiding inadvertent removal of genuine biological differences. Moreover, batch correction requires knowing what the batches are, and that they are at least partially independent of the biological differences, and therefore may not always be feasible.

Comparative analyses against existing methods that can compute pathway activity per cell—AUCell, ssGSEA, and VAM—demonstrate SiPSiC's superior ability to identify real, biologically meaningful results while substantially reducing computational execution time. In summary, we demonstrate that SiPSiC provides accurate, comprehensive, and useful insights into biological pathway activity at the single-cell level. We attribute this success to the combination of proper normalization, ensuring comparable contribution from different genes, and rank-based weighting, enabling the utilization of the higher information content of highly expressed genes.

However, our algorithm does present potential pitfalls. First, SiPSiC's results depend on the units in which gene expression data are provided. Based on our analyses, we recommend providing SiPSiC with TPM values (or CPM if normalization by gene length is not required) rather than logarithmic transformations of these values. Second, SiPSiC is susceptible to outliers, and we therefore recommend filtering both genes and cells before applying this method. Lastly, the activation of many biological processes involves the induction of some genes and the silencing of others. SiPSiC does not account for genes changing in the opposite direction in the same gene set, hence gene sets used as input for SiPSiC analysis should be separated according to the direction of change upon activation of the pathway. To address this, we selected the MSigDB hallmark pathway database, whose pathways meet this criterion (Liberzon et al. 2015). Although other methods developed for bulk RNA-seq data can model more complex pathway structures (Tarca et al. 2009; Vaske et al. 2010; Drier et al. 2013; Young and Craft 2016), they rely on high-quality data and therefore are prone to errors when applied on sparse and noisy data typically achieved by single-cell RNA-seq.

## Methods

### The SiPSiC algorithm

Taking an scRNA-seq gene expression matrix *X* in TPM or CPM and taking a given gene set, SiPSiC performs the following steps to calculate the score for all the cells in the data:

**(1) Calculate normalized gene scores.** Calculate the median of the τ percentage of cells with highest expression (default τ = 5%). If it is positive, use it as normalization factor; if zero, use the maximum value as the normalization factor. Calculate new normalized gene scores for each gene *i* in cell *j:* $S_{i,j}=\frac{X_{i,j}}{NF_{i}}$, where *NF*_*i*_ is the normalization factor.

The reason behind this step is that scRNA-seq data are normally sparse (Chi and Deng 2020); namely, the fraction of zeros in the data is large. Thus, by selecting the median of the top τ% cells, there is a high likelihood that for most genes the value will be greater than zero, but still not be an outlier, which could perturb further processing steps.

**(2) Pathway scoring.** Rank the genes in the gene set by their total expression across all cells $\sum_{j}X_{i,j}$. The pathway score P_j_ is the weighted average of the normalized gene scores by the normalized rank $\left(\frac{rank_{i}}{n_{P}}\right)$: $P_{j}=\frac{1}{{n_{P}}^{2}}*\sum_{i=1}^{n_{P}}rank_{i}*S_{i,j}$.

### scRNA-seq data set preprocessing

We downloaded published data sets from five papers (Tirosh et al. 2016; Neftel et al. 2019; Kim et al. 2020; Melms et al. 2021; Speranza et al. 2021). For the human COVID-19, lung adenocarcinoma, and oligodendroglioma data sets, cell identity annotations were downloaded as well. For the glioblastoma Smart-seq2 data set, we used the malignant cell metamodule assignment that was published. To identify cell identities for the monkey COVID-19 data set, we first identified biomarkers for alveolar, activated B, and CD8^+^ T cells. First, we used the human COVID-19 data set to extract markers of each cell type compared with all other cells, using the FindMarkers function of Seurat (Butler et al. 2018), with logfc.threshold = 1 and min.diff.pct = 0.5, following the parameters used by Speranza et al. (2021). In addition to these markers, we also selected canonical marker genes of alveolar, B, and T cells from the literature: *CAV1*, *PDPN*, *SFTPB*, *SFTPC*, and *SFTPD* were used for alveolar cells (Chen et al. 2004); *MS4A1 (CD20*), *CD27*, and *CD28* for B cells (Sanz et al. 2019); and *CD2*, *CD8A*, and *CXCR3* (*CD138*) for T cells (Tzankov et al. 2005; Groom and Luster 2011). Next, we clustered the monkey data set using R's Seurat clustering pipeline described below with a 0.5 resolution (for the findClusters function), and all clusters were annotated as a given cell type if and only if they displayed high expression of both the canonical markers and the markers identified in the human data set. This resulted in 1411, 1060, and 17,365 cells identified as alveolar, B, and CD8^+^ T cells, respectively, before filtering was applied. After filtering, these numbers dropped to 1004 (86 control; 217, 3 days; and 701, 10 days) alveolar cells, 113 (29 control; 71, 3 days; and 13, 10 days) B cells, and 2224 (360 control; 1162, 3 days; and 702, 10 days) T cells.

The glioblastoma Smart-seq2, lung adenocarcinoma, and oligodendroglioma data sets were log normalized by the original authors; hence, we first converted them to linear scale. For the glioblastoma 10x Genomics data set, we used all malignant cells and genes reported by Neftel et al. (2019) after their filtering. For all other data sets, we took all relevant cells (see below), removed cells expressing (at least one read) less than 1000 genes, and then excluded genes expressed in <10% of the remaining cells. For the two COVID-19 data sets, we selected alveolar cells, B cells, and CD8^+^ T cells, and applied the above filtering for each cell type separately. Because both data sets were supplied as simple count matrices, we divided all transcripts by the total counts of their cell of origin and multiplied by 1 million after filtering. For the lung adenocarcinoma data set, we selected only tS1, tS2, and tS3 cells. For oligodendroglioma, we selected only malignant cells.

### SiPSiC score comparisons

We applied SiPSiC to each data set separately. An unpaired Student's *t*-test was used to compare SiPSiC scores between two groups of cells. When more than two groups of cells were identified in the data set, we performed all pairwise comparisons. The reported FDR values represent the largest FDR of all pairwise comparisons. We considered a pathway upregulated or downregulated only when there was, respectively, a positive or negative median difference and an FDR < 0.01 compared with each of the other groups, unless stated otherwise.

To infer that the Mo et al. (2020) hypoxia signature was upregulated in the tS2 lineage, we relied on the fact that all four genes included in this signature were tS2 specifc (Supplementary Data 3 of Kim et al. 2020). In the glioblastoma data set analysis, because Garofano et al. (2021) performed pathway analysis on different groups of cells than the ones reported in Neftel et al. (2019), we relied on the overlaps between the two classifications. Because the PPR and NEU clusters were enriched in both NPC- and OPC-like cells, when a specific pathway was reported as upregulated in one of these clusters, we considered our result consistent with the findings of Garofano et al. (2021) even when only one of the respective cell states (either OPC- or NPC-like) showed upregulation of that pathway compared with the MES- and AC-like cell states. Such “semiconsistent” pathways account for five of the 31 consistent pathways mentioned in the results section.

Heatmaps were generated using the R package pheatmap, version 1.0.12. Only differential pathways are shown. After selecting relevant cell groups (see below), we calculated *Z*-scores from SiPSiC pathway scores for each of the pathways individually. We sorted the cells in each cell group based on their average *Z*-score across all pathways upregulated in that group and plotted them in ascending order (left to right). The pathways upregulated in each group were sorted in ascending order of FDR values (most significant result at the top). Following the emphasis in the main text, only the active infection (3 day) and control groups were selected for the monkey COVID-19 data set, whereas for the lung adenocarcinoma data set, we only compared the tS1 and tS2 cell lineages, as the tS3 lineage contained only 63 cells. Because almost all pathways showed a significant difference between these two groups, we included the 20 most significant pathways in the heatmap and sorted the cells in each of the two lineages according to their average *Z*-scores across these 20 pathways.

### Clustering and cluster composition analysis

We identified malignant cells in the 10x Genomics data set published by Neftel et al. (2019), and clustered them using the Louvain algorithm implemented in R's Seurat package, both based on single genes and on SiPSiC pathway scores. Cell annotations were based on the markers provided by Neftel et al. (2019) for the malignant metamodules they defined. Batch correction was applied by following the standard pipeline described in the documentation of Seurat, scVI, and Harmony. For additional details, see the Supplemental Methods.

### Method benchmarking

We repeated the SiPSiC analyses for the glioblastoma (Smart-seq2) and two COVID-19 data sets using AUCell version 1.24.0 and VAM 1.1.0. ssGSEA was applied using its implementation in R's GSVA package (Hänzelmann et al. 2013), version 1.50.0. To guarantee integrity of the results, the same preprocessing steps were applied; cell assignments to the different groups were kept; and pathway up- or downregulation was defined as in our SiPSiC analyses across the data sets. All parameters of the different methods were set to default. Pathways skipped by AUCell but consistently detected by all other methods as either up- or downregulated in a specific group were counted both as skipped and missed by AUCell.

Violin plots were generated with the R package ggplot2, version 3.4.4. For each method, all cell scores were normalized with (divided by) the maximum score produced by this method for the relevant pathway prior to plot generation.

We used R studio's profiler (R's Profvis package, version 0.3.8) to record the execution of the different methods on a computer with Windows 11 x64, 6 Intel i7-8700 (3.20 GHz) cores and 64 GB RAM. The R version was 4.3.2 (R Core Team 2023) used in R studio version 2023.12.0 (build 369). SiPSiC execution times were calculated as the sum of all calls to the getPathwayScores function for each of the 50 hallmark pathways, whereas for AUCell, we measured the execution time of the AUCell_run function. Because these functions also include the detection of the relevant pathway genes in the input data matrix, for VAM, we summed the executions of the createGeneSetCollection and vamForCollection functions, whereas for ssGSEA, the ssgseaParam function was ignored as its execution time never passed R studio's profiler threshold for detection (20 msec), and only the call to the gsva function was considered.

### Software availability

SiPSiC is available at Bioconductor (https://bioconductor.org/packages/SiPSiC), at GitHub (https://github.com/DanielDavis12/SiPSiC), and as Supplemental Code 1. Custom scripts applying SiPSiC and the other methods to the different data sets are available as Supplemental Code 2.

## Supplementary Material

Supplement 1

Supplement 2

Supplement 3

Supplement 4

Supplement 5

Supplement 6

Supplement 7

Supplement 8

Supplement 9

Supplement 10

Supplement 11
