# Supplementary material for: Accurate estimation of pathway activity in single cells for clustering and differential analysis
Source: Genome Res. 2024 Jun;34(6):925–36. doi: 10.1101/gr.278431.123 (PMC11293543; doi:10.1101/gr.278431.123)
Supplement: Supplement 1 [file Supplemental_Materials.pdf]

Supplemental Materials for

Accurately estimating pathway activity in single cells for clustering and differential analysis

Daniel Davis, Avishai Wizel, Yotam Drier

The Lautenberg Center for Immunology and Cancer Research, IMRIC, Faculty of Medicine, Hebrew University of Jerusalem, Israel.

Contents

Supplemental Figures S1 – S3
Supplemental Note 1: SiPSiC differential pathway analysis of the African green monkey COVID-19 dataset
Supplemental Note 2: Comparison of SiPSiC pathway analysis of the glioblastoma dataset to the reported differential pathway activity from Garofano et al.
Supplemental Note 3: SiPSiC differential pathway analysis of the oligodendroglioma dataset
Supplemental Methods: Clustering and cluster composition analysis
References

Additional supplemental materials for this paper:

Supplemental Table S1: Complete differential pathway analysis results of the human COVID dataset by SiPSiC, AUCell, ssGSEA and VAM
Supplemental Table S2: Complete differential pathway analysis results of the monkey COVID dataset by SiPSiC, AUCell, ssGSEA and VAM
Supplemental Table S3: Complete SiPSiC differential pathway analysis results of the lung adenocarcinoma dataset
Supplemental Table S4: Complete differential pathway analysis results of the glioblastoma dataset by SiPSiC, AUCell, ssGSEA and VAM
Supplemental Table S5: Complete SiPSiC differential pathway analysis results of the oligodendroglioma dataset
Supplemental Table S6: Differentially active pathways in glioblastoma cell clusters calculated for the malignant cells of the glioblastoma 10x Genomics dataset
Supplemental Table S7: Execution times of SiPSiC, AUCell, ssGSEA and VAM for the hallmark pathway enrichment analysis of the glioblastoma and both COVID datasets
Supplemental Table S8: Summary of SiPSiC results for differential pathway analysis with different values of τ

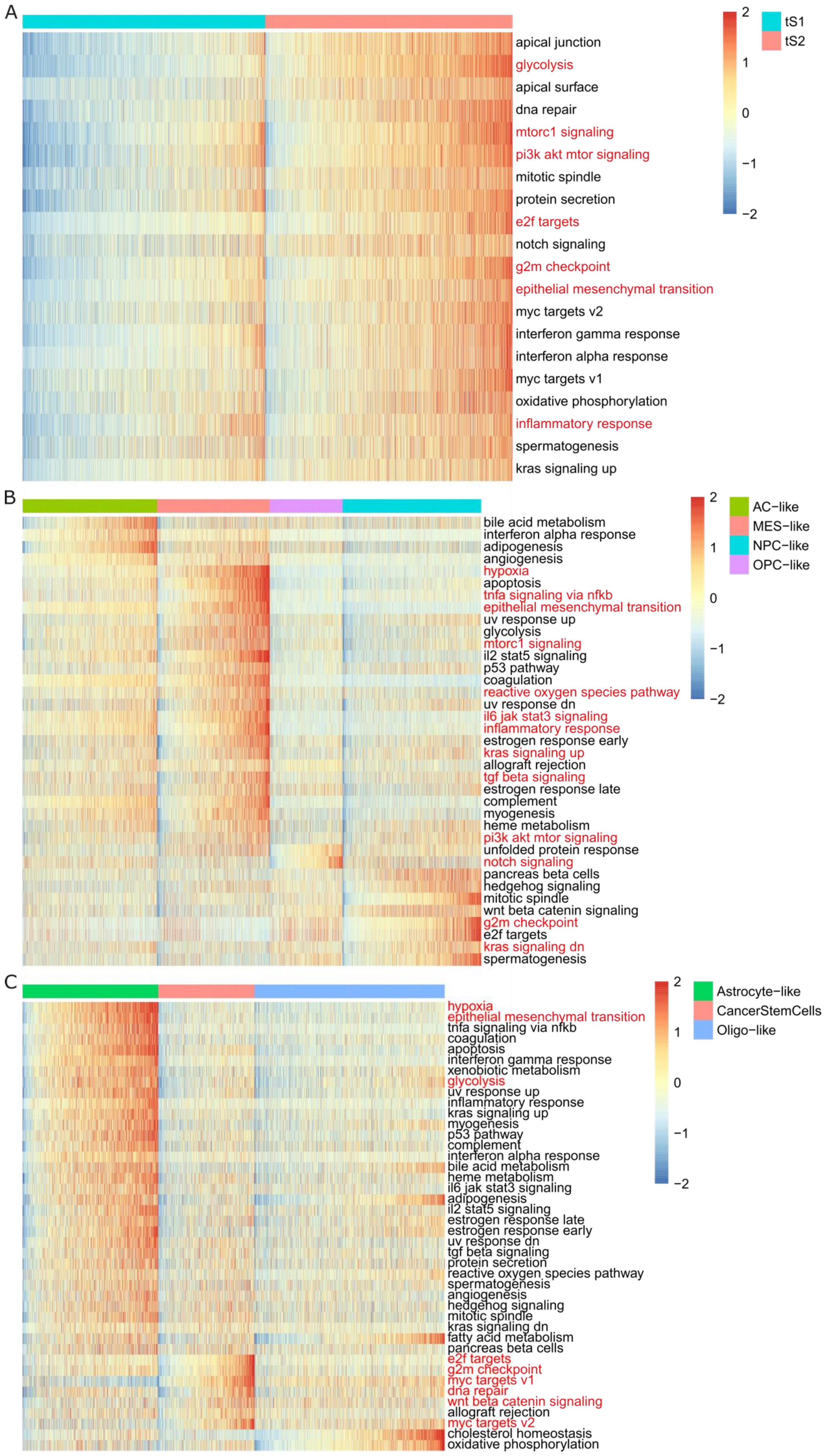
A
tS1
tS2
2
1
0
−1
−2
apical junction
glycolysis
apical surface
dna repair
mtorc1 signaling
pi3k akt mtor signaling
mitotic spindle
protein secretion
e2f targets
notch signaling
g2m checkpoint
epithelial mesenchymal transition
myc targets v2
interferon gamma response
interferon alpha response
myc targets v1
oxidative phosphorylation
inflammatory response
spermatogenesis
kras signaling up
B
AC−like
MES−like
NPC−like
OPC−like
bile acid metabolism
interferon alpha response
adipogenesis
angiogenesis
hypoxia
apoptosis
tnfa signaling via nfkb
epithelial mesenchymal transition
uv response up
glycolysis
mtorc1 signaling
il2 stat5 signaling
p53 pathway
coagulation
reactive oxygen species pathway
uv response dn
il6 jak stat3 signaling
inflammatory response
estrogen response early
kras signaling up
allograft rejection
tgf beta signaling
estrogen response late
complement
myogenesis
heme metabolism
pi3k akt mtor signaling
unfolded protein response
notch signaling
pancreas beta cells
hedgehog signaling
mitotic spindle
wnt beta catenin signaling
g2m checkpoint
e2f targets
kras signaling dn
spermatogenesis
C
Astrocyte−like
CancerStemCells
Oligo−like
hypoxia
epithelial mesenchymal transition
tnfa signaling via nfkb
coagulation
apoptosis
interferon gamma response
xenobiotic metabolism
glycolysis
uv response up
inflammatory response
kras signaling up
myogenesis
p53 pathway
complement
interferon alpha response
bile acid metabolism
heme metabolism
il6 jak stat3 signaling
adipogenesis
il2 stat5 signaling
estrogen response late
estrogen response early
uv response dn
tgf beta signaling
protein secretion
reactive oxygen species pathway
spermatogenesis
angiogenesis
hedgehog signaling
mitotic spindle
kras signaling dn
fatty acid metabolism
pancreas beta cells
e2f targets
g2m checkpoint
myc targets v1
dna repair
wnt beta catenin signaling
allograft rejection
myc targets v2
cholesterol homeostasis
oxidative phosphorylation

Supplemental Fig. S1: SiPSiC detects differential pathway activity of malignant subclones across cancer types. **(A)** Heatmap depicting *Z*-scores of SiPSiC scores of the 20 most differential hallmark pathways in lung adenocarcinoma. Pathways are sorted by significance of differential scores, cells in each cell group are sorted by their average *Z*-score across all shown pathways. Pathway names mentioned in the text are colored red. **(B)** Heatmap depicting *Z*-scores of SiPSiC scores of all differential hallmark pathways (FDR < 0.01) in glioblastoma. Pathways upregulated in each group are sorted by significance of differential scores, cells in each cell group are sorted by their average *Z*-score across all pathways upregulated in that group. Pathway names mentioned in the text are in red. **(C)** Heatmap depicting *Z*-scores of SiPSiC scores of all differential hallmark pathways (FDR < 0.01) in oligodendroglioma. Pathways upregulated in each group are sorted by significance of differential scores, cells in each cell group are sorted by their average *Z*-score across all pathways upregulated in that group. Pathway names mentioned in the text are in red.

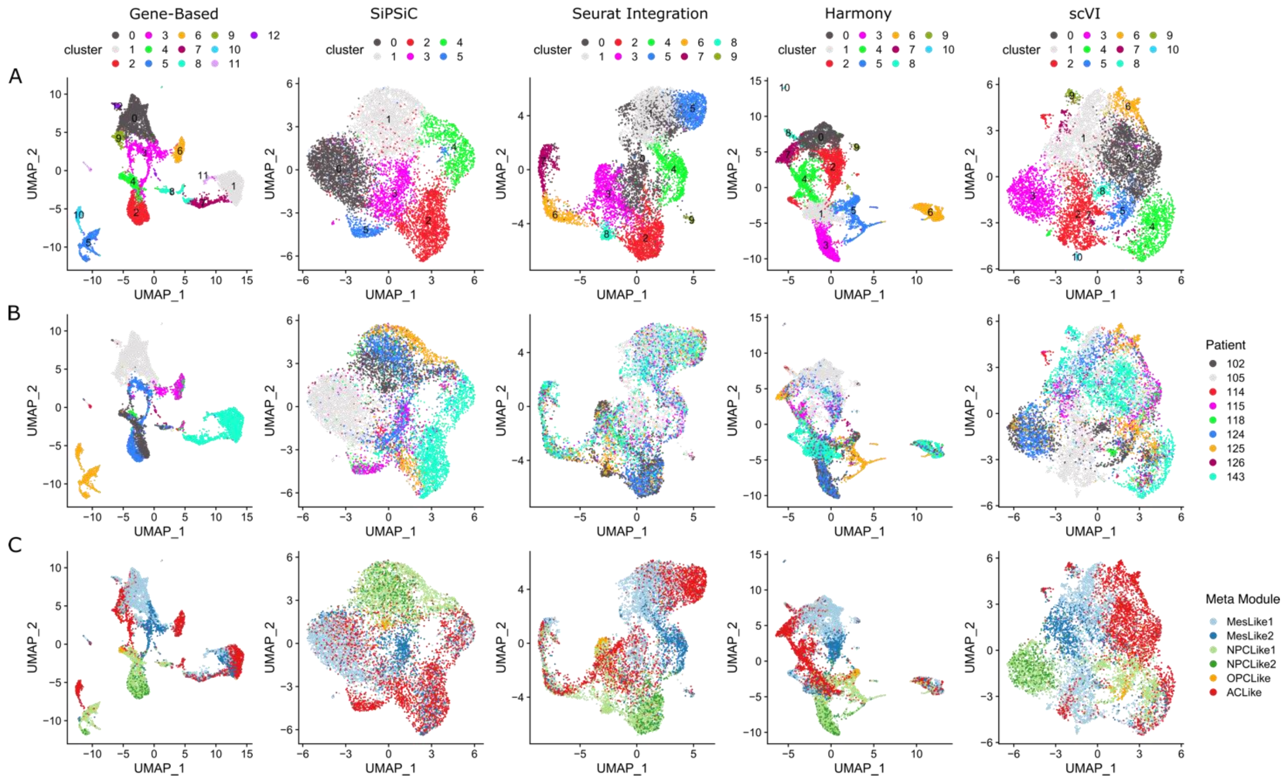

Supplemental Fig. S2: SiPSiC's ability to overcome patient batch effects is comparable to explicit batch correction methods. **(A-C)** UMAP projections based on gene expression, SiPSiC scores, Seurat Integration, Harmony and scVI, as shown in the titles on top. **(A)** Cells were clustered by Louvain algorithm according to gene expression, SiPSiC scores, Seurat Integration, Harmony and scVI, as shown in the titles on top, UMAPs show cells colored by cluster. **(B)** Cells colored by patient identity. **(C)** Cells colored by malignant meta-module assignment.

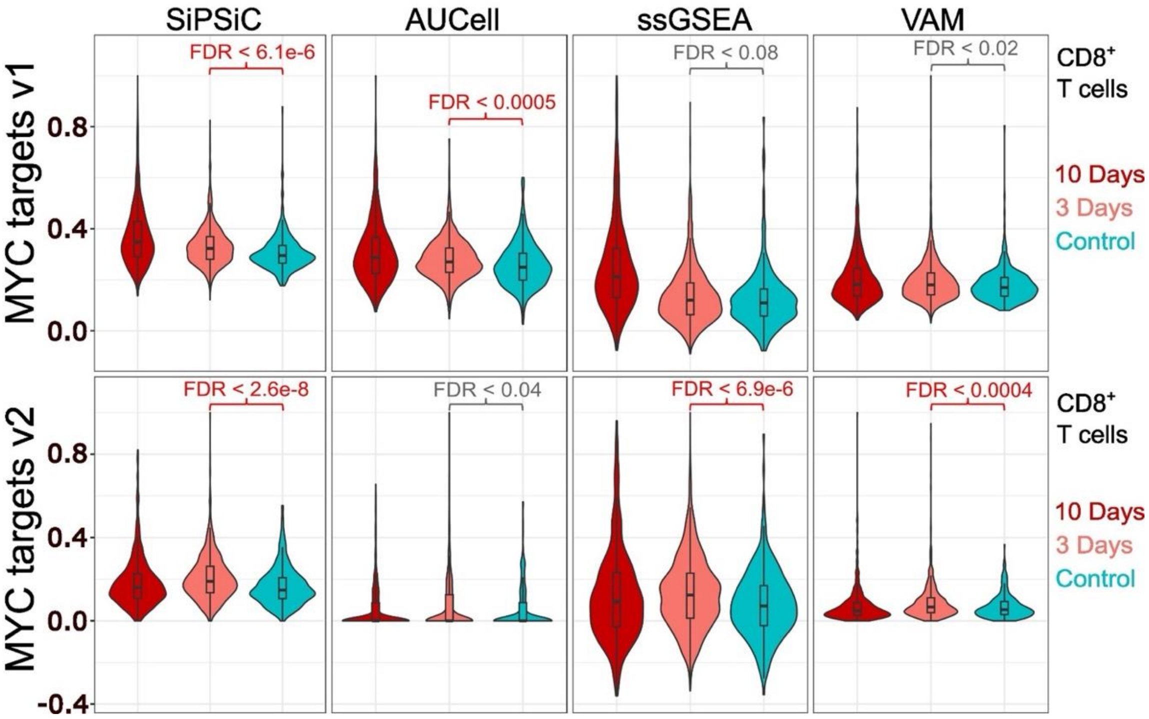

Supplemental Fig. S3: SiPSiC consistently detects upregulation of MYC in CD8$^+$ T Cells of SARS-CoV-2 infected monkeys. Violin plots showing normalized pathway scores distribution of the MYC targets pathways for the monkey CD8$^+$ T cells, as calculated by the four different methods. Significant results (FDR < 0.01) are in red.

Supplemental Note 1 - SiPSiC differential pathway analysis of the African green monkey COVID-19 dataset:

In the African green monkey experiment, two monkeys were inoculated with inactivated SARS-CoV-2 (henceforth referred to as control) and eight with the active virus (Speranza et al., 2021). To better study the dynamics of viral infection, these eight monkeys were then split into two groups of four monkeys each and euthanized three (first group) or ten (second group) days post inoculation; the monkeys in the 10-day group had already recovered by the 10th day. Hence, we focused on comparing the active infection (3-day) to the control after calculating the pathway scores per cell type (Methods). Complete results of all three groups can be found in Supplemental Table S2.

Analysis of this dataset provided support for pathways that were found upregulated from all three categories. We found 20 upregulated and 3 downregulated pathways in alveolar cells (Figure 1B). Here too the cells in the active infection group were enriched in the interferon alpha and gamma response, complement, E2F targets and apoptosis pathways relative to both other groups (Supplemental Table S2), supporting our findings from the analysis of human alveolar cells. In addition, most of the pathways showing therapeutic potential that were found upregulated in the COVID-19 group of alveolar cells in the human dataset were also upregulated in the alveolar cells of the active infection group in the monkey dataset, compared to control. These are the DNA repair, mTORC1 and PI3K/AKT/MTOR signaling, glycolysis, reactive oxygen species and unfolded protein response pathways. Out of all these pathways, only interferon signaling was identified by Speranza et al., further highlighting SiPSiC's high sensitivity.

Analysis of the immune cells that were found in the monkeys' lungs revealed that in both B and $CD8^+$ T cells, the interferon alpha and gamma response pathways were enriched in the 3-day group, in accordance with our finding of interferon gamma upregulation in $CD8^+$ T cells from the human dataset and the evidence showing elevated interferon levels in the plasma of COVID-19 patients and its consequences on immune cells (Schultheiß et al., 2020; Speranza et al., 2021). In addition, both MYC targets pathways (V1 and V2) were found upregulated in the active infection (3-day) group of $CD8^+$ T cells compared to control. We indeed expect to see upregulation of MYC targets in T cells, since MYC is known to be involved in T cell proliferation (Gnanaprakasam & Wang, 2017), and increased T cell proliferation occurs in COVID-19 patients' lungs (Liao et al., 2020). To confirm this finding, we compared *MYC* expression in the control and active infection groups and found that average *MYC* expression 3 days after infection was 89% higher than control ($p < 0.0032$, unpaired Wilcoxon test), strongly supporting SiPSiC's finding. Furthermore, the inflammatory response pathway was also enriched in the 3-day group of $CD8^+$ T cells, reflecting the anti-viral activity.

SiPSiC also demonstrated the upregulation of the IL6/JAK/STAT3 signaling pathway in the active infection group of $CD8^+$ T cells compared to control (FDR < 5.61×10^{-9}), a finding supported by evidence for high levels of IL6 in the serum of COVID-19 patients even in relatively mild cases and increased IL6 secretion from epithelial cells infected with SARS-CoV-2 (Han et al., 2020; Patra et al., 2020; Sanli et al., 2021). This is another example for SiPSiC's potential to highlight therapeutically relevant pathways, as the IL6/JAK/STAT3 signaling pathway has been suggested as a therapeutic target for COVID-19 (Jafarzadeh et al., 2021). Similarly, the Notch signaling pathway was found upregulated in this group, although with borderline statistical significance (FDR < 0.034). Notch too has been suggested as a therapeutic target in COVID-19, as it is known to modulate the immune response and is involved in a positive feedback loop with *IL6* expression in macrophages (Rizzo et al., 2020). This suggests that $CD8^+$ T cells may exhibit the same feedback loop upon SARS-CoV-2 infection, and that IL6 signaling could be moderated in these cells by inhibiting Notch signaling.

Supplemental Note 2 - Comparison of SiPSiC pathway analysis of the glioblastoma dataset to the reported differential pathway activity from Garofano et al.:

Garofano et al. classified the cells into four distinct cell clusters: glycolytic/plurimetabolic (GPM), mitochondrial (MTC), neural (NEU) and proliferative/progenitor (PPR) (Garofano et al., 2021). They found that the GPM and MTC clusters were enriched in cells of the MES- and AC-like cell states, respectively, while the PPR and NEU clusters were enriched in cells of both the OPC- and NPC-like states. Gene set enrichment of these clusters is reported for 40 hallmark pathways, and we compared their results to SiPSiC's. 31 out of the 40 pathways (78%) were consistent, in the sense that if a pathway was reported as upregulated in one of Garofano et al. cell clusters, SiPSiC also found it upregulated in the same cell state this cell cluster is enriched in. For instance, Garofano et al. reported that the DNA repair pathway was upregulated in the PPR cell cluster, which is enriched in both OPC- and NPC-like cells. Indeed, SiPSiC found this pathway was upregulated in both the OPC- and NPC-like cell states compared to both the AC- and MES-like states. Furthermore, out of the nine other pathways, eight were found by Garofano et al. to be enriched in the MTC cluster, hence we expected them to be upregulated in the AC-like cell state in our analysis. Seven of these pathways were found to be "near-consistent" in the sense that they were significantly upregulated in the AC-like cell state compared to two of the other three cell states and insignificantly either up- (5 pathways) or down-regulated (2 pathways) compared to the third other cell state, making 38 of the 40 (95%) pathways reported by Garofano et al. either completely or near consistent in our SiPSiC analysis.

The two remaining pathways are the hallmark Notch and mTORC1 signaling pathways. While Garofano et al. found that the MTC cluster was enriched in the hallmark Notch signaling pathway (reported $FDR < 1.23 \times 10^{-3}$), SiPSiC analysis found that it was upregulated in the OPC-like cell state ($FDR < 0.0095$). Although Neftel et al. did not report upregulation of Notch in any of the cell states, AUCell supported SiPSiC's result finding Notch upregulation in the OPC-like cell state (Supplemental Table S4), but ssGSEA and VAM did not capture this. Prior research has shown that Notch signaling activation inhibits the maturation and may enhance the proliferation of oligodendrocyte progenitor cells (John et al., 2002; C. Wang et al., 2017; S. Wang et al., 1998), suggesting that Notch upregulation in OPC-like glioblastoma cells may play similar roles in the pathophysiology of the disease. Similarly, while Garofano et al. found that the mTORC1 signaling pathway was enriched in the PPR cluster (reported $FDR < 1.26 \times 10^{-3}$) and Neftel et al. did not report its upregulation in any of the cell states, SiPSiC found that it was upregulated in the MES-like cell state ($FDR < 3.1 \times 10^{-75}$), supported by AUCell and VAM which also found the same (Supplemental Table S4). Prior research has shown that in epithelial cells, TGFB1-induced EMT both promotes and requires the activation of the PI3K/AKT/MTOR pathway, and more specifically activates the mTORC1 complex (Lamouille

et al., 2014). The same has also been suggested for an EMT-like process in glioblastoma (Iser et al., 2017; Zhang et al., 2014). SiPSiC found upregulation of the PI3K/AKT/MTOR signaling pathway in the MES-like cell state (supported by AUCell and ssGSEA), in addition to the upregulation of the TGF beta signaling and EMT which were mentioned above (both supported by AUCell, ssGSEA and VAM, see Supplemental Table S4). Combined, these findings may well account for upregulation of the mTORC1 signaling in this cell state, supporting our finding over that reported by Garofano et al. for this pathway.

Supplemental Note 3 - SiPSiC differential pathway analysis of the oligodendroglioma dataset:

As further validation, we applied SiPSiC to a lower-grade oligodendroglioma scRNA-seq dataset (Tirosh et al., 2016). We compared SiPSiC scores of three subpopulations: 924 cancer stem cells (CSC), 1300 astrocyte-like cells and 1820 oligodendrocyte-like cells (Supplemental Table S5 and Supplemental Fig. S1C). SiPSiC identified upregulation of the G2/M checkpoint pathway in the CSC group (FDR < 3.3×10^{-12}), consistent with the findings of Tirosh et al. Furthermore, SiPSiC identified other relevant differential pathways not reported in the original analysis. The Wnt/beta catenin signaling (FDR < 1.6×10^{-6}) and DNA repair pathways (FDR < 1.3×10^{-6}) were upregulated in the CSCs compared to the two other groups, consistent with evidence that Wnt signaling plays a central role in the maintenance of stem cells in several tissues and the stemness of glioma cells in particular (Fodde & Brabletz, 2007; Jin et al., 2011; Zheng et al., 2010) and that in glioma stem cells, activation of the DNA damage response confers radioresistance (Bao et al., 2006). Additionally, the CSC subpopulation showed upregulation of the E2F targets (FDR < 1.5×10^{-26}) and MYC targets pathways (FDR < 4.9×10^{-12} for MYC targets V1 and FDR < 0.0009 for MYC targets V2). *MYC* is more highly expressed in glioma CSCs compared to non-stem glioma cells, and knockdown of *MYC* reduces CSC proliferation and promotes apoptosis (J. Wang et al., 2008). Lymphoid-specific helicase (HELLS) activity is essential for glioblastoma stem cells and correlates with MYC and E2F targets (G. Zhang et al., 2019), suggesting SiPSiC captures elevated HELLS activity in CSCs.

Furthermore, SiPSiC analysis suggested that the astrocyte-like cells in oligodendroglioma also harbor a mesenchymal phenotype. 20 out of 23 pathways (87%) that were upregulated in the MES-like glioblastoma cells mentioned above were also upregulated in the astrocyte-like cells of oligodendroglioma, including EMT, hypoxia, glycolysis, and several inflammation-related pathways, a significant overlap (p < 0.005, Fisher's exact test). Similarly, all four pathways upregulated in the AC-like glioblastoma cells were also upregulated in the astrocyte-like cells of oligodendroglioma, providing further evidence of their astrocytic nature.

Supplemental Methods - Clustering and cluster composition analysis:

We started with all gene expression data provided in the glioblastoma 10x Genomics dataset published by Neftel et al. To distinguish between malignant and non-malignant cells, we first inferred malignant cell markers from the Smart-seq2 dataset according to the cell annotation provided by Neftel et al. Markers were calculated using the Seurat FindMarkers function with all parameters set to default, and the 10 genes with the highest fold change that passed the adjusted $p < 0.01$ threshold were selected as the final malignant markers. This resulted in the following genes: *PTPRZ1*, *IGF2*, *FABP7*, *GPM6A*, *CHI3L1*, *EGFR*, *IGFBP3*, *BCAN*, *NNAT* and *PDGFRA*. We then calculated for each cell in the 10x Genomics dataset the average expression (in log(TPM +1) units) of these 10 markers as well as the markers of T cells, macrophages and non-malignant oligodendrocytes provided by Neftel et al. We clustered all cells using the Louvain algorithm by applying the Seurat FindClusters function with a 0.5 resolution using the first 15 principal components. This yielded 12 clusters, each showing either upregulation of malignant markers or markers of one of the non-malignant cell types. For downstream analysis we focused on the 9635 cells in the clusters marked as malignant.

These cells were then clustered twice using Seurat (version 4.4.0) with identical parameters, first based on gene expression and second based on SiPSiC's pathway scores. The first 15 principal components were identified using RunPCA, and used to find the 20-nearest neighbors using FindNeighbors (with dims = 15). Cells were clustered by the Louvain algorithm as implemented in the FindClusters function with a resolution of 0.3. Differential pathways were identified in the pathway-based clustering, using the Wilcoxon rank sum test implemented in Seurat's FindAllMarkers function, using parameters slot = counts, only.pos = true and logfc.threshold = 0.03. All UMAP projections were calculated by RunUMAP based on the first 15 principal components.

We used the marker genes provided by Neftel et al. for each malignant meta-module to annotate the cells, using the Seurat addModuleScore function with ctrl = 50, assigning each cell to the meta-module with the maximal score. This annotation resulted in 2919 AC-like, 2000 NPC-like1, 527 NPC-like2, 2869 MES-like1, 1148 MES-like2 and 172 OPC-like cells. Throughout the clustering section we used the terms NPC- or MES-like cells to refer to the combination NPC-like1 and NPC-like2 cells or MES-like1 and MES-like2 cells, respectively.

To integrate the data for batch correction using Seurat we implemented the standard Seurat integration pipeline. We started by finding the 2000 most variable genes in each patient's normalized data using the SplitObject, NormalizeData and FindVariableFeatures functions, then detected the relevant features for integration using the SelectIntegrationFeatures and FindIntegrationAnchors functions with default values. Lastly, we integrated the data by executing the IntegrateData function with k.weight = 70. We then clustered the cells using the same functions and parameters described above for the clustering based on SiPSiC scores.

To apply the scVI integration method, we followed the scVI documentation and used the raw counts of the same 10x Genomics dataset rather than the TPM values we used in all other integration methods. Integration was applied using the Python package scVI-tools, version 1.1.2, and Python version 3.9.18. We first selected the 2000 most variable genes using the FindVariableFeatures function, then followed the standard scVI pipeline by calling the setup_anndata function with batch_key set to the patient identity as well as the scVI and train functions to create and train the model required for integration. We proceeded with calling the get_latent_representation function then the Seurat CreateDimReducObject, and finally executed the FindNeighbors and RunUMAP functions with dims = 1:10 and the scVI reduction as input for the reduction parameter, as instructed by the scVI documentation. For the Harmony integration we used the R harmony package version 1.2.0. We executed the RunHarmony function with the patient identity variable name as input and all other values set to default. We then clustered the cells using the same functions and parameters described above for the clustering based on SiPSiC scores.

References

Bao, S., Wu, Q., McLendon, R. E., Hao, Y., Shi, Q., Hjelmeland, A. B., Dewhirst, M. W., Bigner, D. D., & Rich, J. N. (2006). Glioma stem cells promote radioresistance by preferential activation of the DNA damage response. *Nature*, *444*(7120), 756–760. https://doi.org/10.1038/nature05236

Fodde, R., & Brabletz, T. (2007). Wnt/β-catenin signaling in cancer stemness and malignant behavior. *Current Opinion in Cell Biology*, *19*(2), 150–158. https://doi.org/10.1016/j.ceb.2007.02.007

Garofano, L., Migliozzi, S., Oh, Y. T., D'Angelo, F., Najac, R. D., Ko, A., Frangaj, B., Caruso, F. P., Yu, K., Yuan, J., Zhao, W., Luisa Di Stefano, A., Bielle, F., Jiang, T., Sims, P., Suvà, M. L., Tang, F., Su, X. D., Ceccarelli, M., … Iavarone, A. (2021). Pathway-based classification of glioblastoma uncovers a mitochondrial subtype with therapeutic vulnerabilities. *Nature Cancer*, *2*(2), 141–156. https://doi.org/10.1038/s43018-020-00159-4

Gnanaprakasam, J. N., & Wang, R. (2017). MYC in Regulating Immunity: Metabolism and Beyond. *Genes*, *8*(3), 88. https://doi.org/10.3390/genes8030088

Han, H., Ma, Q., Li, C., Liu, R., Zhao, L., Wang, W., Zhang, P., Liu, X., Gao, G., Liu, F., Jiang, Y., Cheng, X., Zhu, C., & Xia, Y. (2020). Profiling serum cytokines in COVID-19 patients reveals IL-6 and IL-10 are disease severity predictors. *Emerging Microbes & Infections*, *9*(1), 1123–1130. https://doi.org/10.1080/22221751.2020.1770129

Iser, I. C., Pereira, M. B., Lenz, G., & Wink, M. R. (2017). The Epithelial-to-Mesenchymal Transition-Like Process in Glioblastoma: An Updated Systematic Review and In Silico Investigation. *Medicinal Research Reviews*, *37*(2), 271–313. https://doi.org/10.1002/med.21408

Jafarzadeh, A., Nemati, M., & Jafarzadeh, S. (2021). Contribution of STAT3 to the pathogenesis of COVID-19. *Microbial Pathogenesis*, *154*, 104836. https://doi.org/10.1016/j.micpath.2021.104836

Jin, X., Jeon, H.-Y., Joo, K. M., Kim, J.-K., Jin, J., Kim, S. H., Kang, B. G., Beck, S., Lee, S. J., Kim, J. K., Park, A.-K., Park, W.-Y., Choi, Y.-J., Nam, D.-H., & Kim, H. (2011). Frizzled 4 Regulates Stemness and Invasiveness of Migrating Glioma Cells Established by Serial Intracranial Transplantation. *Cancer Research*, *71*(8), 3066–3075. https://doi.org/10.1158/0008-5472.CAN-10-1495

John, G. R., Shankar, S. L., Shafit-Zagardo, B., Massimi, A., Lee, S. C., Raine, C. S., & Brosnan, C. F. (2002). Multiple sclerosis: Re-expression of a developmental pathway that restricts oligodendrocyte maturation. *Nature Medicine*, *8*(10), 1115–1121. https://doi.org/10.1038/nm781

Lamouille, S., Xu, J., & Derynck, R. (2014). Molecular mechanisms of epithelial–mesenchymal transition. *Nature Reviews Molecular Cell Biology*, *15*(3), 178–196. https://doi.org/10.1038/nrm3758

Liao, M., Liu, Y., Yuan, J., Wen, Y., Xu, G., Zhao, J., Cheng, L., Li, J., Wang, X., Wang, F., Liu, L., Amit, I., Zhang, S., & Zhang, Z. (2020). Single-cell landscape of bronchoalveolar immune cells in patients with COVID-19. *Nature Medicine*, *26*(6), 842–844. https://doi.org/10.1038/s41591-020-0901-9

Patra, T., Meyer, K., Geerling, L., Isbell, T. S., Hoft, D. F., Brien, J., Pinto, A. K., Ray, R. B., & Ray, R. (2020). SARS-CoV-2 spike protein promotes IL-6 trans-signaling by activation of angiotensin II receptor signaling in epithelial cells. *PLOS Pathogens*, *16*(12), e1009128. https://doi.org/10.1371/journal.ppat.1009128

Rizzo, P., Vieceli Dalla Sega, F., Fortini, F., Marracino, L., Rapezzi, C., & Ferrari, R. (2020). COVID-19 in the heart and the lungs: could we “Notch” the inflammatory storm? *Basic Research in Cardiology*, *115*(3), 31. https://doi.org/10.1007/s00395-020-0791-5

Sanli, D. E. T., Altundag, A., Kandemirli, S. G., Yildirim, D., Sanli, A. N., Saatci, O., Kirisoglu, C. E., Dikensoy, O., Murrja, E., Yesil, A., Bastan, S., Karsidag, T., Akinci, I. O., Ozkok, S., Yilmaz, E., Tuzuner, F., Kilercik, M., & Ljama, T. (2021). Relationship between disease severity and serum IL-6 levels in COVID-19 anosmia. *American Journal of Otolaryngology*, *42*(1), 102796. https://doi.org/10.1016/j.amjoto.2020.102796

Schultheiß, C., Paschold, L., Simnica, D., Mohme, M., Willscher, E., von Wenserski, L., Scholz, R., Wieters, I., Dahlke, C., Tolosa, E., Sedding, D. G., Ciesek, S., Addo, M., & Binder, M. (2020). Next-Generation Sequencing of T and B Cell Receptor Repertoires from COVID-19 Patients Showed Signatures Associated with Severity of Disease. *Immunity*, *53*(2), 442-455.e4. https://doi.org/10.1016/J.IMMUNI.2020.06.024

Speranza, E., Williamson, B. N., Feldmann, F., Sturdevant, G. L., Pérez-Pérez, L., Meade-White, K., Smith, B. J., Lovaglio, J., Martens, C., Munster, V. J., Okumura, A., Shaia, C., Feldmann, H., Best, S. M., & de Wit, E. (2021). Single-cell RNA sequencing reveals SARS-CoV-2 infection dynamics in lungs of African green monkeys. In *Sci. Transl. Med* (Vol. 13). http://stm.sciencemag.org/

Tirosh, I., Venteicher, A. S., Hebert, C., Escalante, L. E., Patel, A. P., Yizhak, K., Fisher, J. M., Rodman, C., Mount, C., Filbin, M. G., Neftel, C., Desai, N., Nyman, J., Izar, B., Luo, C. C., Francis, J. M., Patel, A. A., Onozato, M. L., Riggi, N., … Suvà, M. L. (2016). Single-cell RNA-seq supports a developmental hierarchy in human oligodendroglioma. *Nature*, *539*(7628), 309–313. https://doi.org/10.1038/nature20123

Wang, C., Zhang, C.-J., Martin, B. N., Bulek, K., Kang, Z., Zhao, J., Bian, G., Carman, J. A., Gao, J., Dongre, A., Xue, H., Miller, S. D., Qian, Y., Hambardzumyan, D., Hamilton, T., Ransohoff, R. M., & Li, X. (2017). IL-17 induced NOTCH1 activation in oligodendrocyte

progenitor cells enhances proliferation and inflammatory gene expression. *Nature Communications*, *8*(1), 15508. https://doi.org/10.1038/ncomms15508

Wang, J., Wang, H., Li, Z., Wu, Q., Lathia, J. D., McLendon, R. E., Hjelmeland, A. B., & Rich, J. N. (2008). c-Myc Is Required for Maintenance of Glioma Cancer Stem Cells. *PLoS ONE*, *3*(11), e3769. https://doi.org/10.1371/journal.pone.0003769

Wang, S., Sdrulla, A. D., diSibio, G., Bush, G., Nofziger, D., Hicks, C., Weinmaster, G., & Barres, B. A. (1998). Notch Receptor Activation Inhibits Oligodendrocyte Differentiation. *Neuron*, *21*(1), 63–75. https://doi.org/10.1016/S0896-6273(00)80515-2

Zhang, G., Dong, Z., Prager, B. C., Kim, L. J. K., Wu, Q., Gimple, R. C., Wang, X., Bao, S., Hamerlik, P., & Rich, J. N. (2019). Chromatin remodeler HELLS maintains glioma stem cells through E2F3 and MYC. *JCI Insight*, *4*(7). https://doi.org/10.1172/jci.insight.126140

Zhang, L., Wang, H., Zhu, J., Ding, K., & Xu, J. (2014). FTY720 reduces migration and invasion of human glioblastoma cell lines via inhibiting the PI3K/AKT/mTOR/p70S6K signaling pathway. *Tumor Biology*, *35*(11), 10707–10714. https://doi.org/10.1007/s13277-014-2386-y

Zheng, H., Ying, H., Wiedemeyer, R., Yan, H., Quayle, S. N., Ivanova, E. V., Paik, J.-H., Zhang, H., Xiao, Y., Perry, S. R., Hu, J., Vinjamoori, A., Gan, B., Sahin, E., Chheda, M. G., Brennan, C., Wang, Y. A., Hahn, W. C., Chin, L., & DePinho, R. A. (2010). PLAGL2 Regulates Wnt Signaling to Impede Differentiation in Neural Stem Cells and Gliomas. *Cancer Cell*, *17*(5), 497–509. https://doi.org/10.1016/j.ccr.2010.03.020
